# Automatic Identification of Physical Activity Type and Duration by Wearable Activity Trackers: A Validation Study

**DOI:** 10.2196/13547

**Published:** 2019-05-23

**Authors:** Diana Dorn, Jessica Gorzelitz, Ronald Gangnon, David Bell, Kelli Koltyn, Lisa Cadmus-Bertram

**Affiliations:** 1 Department of Kinesiology University of Wisconsin-Madison Madison, WI United States; 2 Department of Population Health Sciences University of Wisconsin-Madison Madison, WI United States

**Keywords:** fitness trackers, exercise, accelerometry, data accuracy

## Abstract

**Background:**

Activity trackers are now ubiquitous in certain populations, with potential applications for health promotion and monitoring and chronic disease management. Understanding the accuracy of this technology is critical to the appropriate and productive use of wearables in health research. Although other peer-reviewed validations have examined other features (eg, steps and heart rate), no published studies to date have addressed the accuracy of automatic activity type detection and duration accuracy in wearable trackers.

**Objective:**

The aim of this study was to examine the ability of 4 commercially available wearable activity trackers (Fitbits Flex 2, Fitbit Alta HR, Fitbit Charge 2, and Garmin Vívosmart HR), in a controlled setting, to correctly and automatically identify the type and duration of the physical activity being performed.

**Methods:**

A total of 8 activity types, including walking and running (on both a treadmill and outdoors), a run embedded in walking bouts, elliptical use, outdoor biking, and pool lap swimming, were tested by 28 to 34 healthy adult participants (69 total participants who participated in some to all activity types). Actual activity type and duration were recorded by study personnel and compared with tracker data using descriptive statistics and mean absolute percent error (MAPE).

**Results:**

The proportion of trials in which the activity type was correctly identified was 93% to 97% (depending on the tracker) for treadmill walking, 93% to 100% for treadmill running, 36% to 62% for treadmill running when preceded and followed by a walk, 97% to 100% for outdoor walking, 100% for outdoor running, 3% to 97% for using an elliptical, 44% to 97% for biking, and 87.5% for swimming. When activities were correctly identified, the MAPE of the detected duration versus the actual activity duration was between 7% and 7.9% for treadmill walking, 8.7% and 144.8% for treadmill running, 23.6% and 28.9% for treadmill running when preceded and followed by a walk, 4.9% and 11.8% for outdoor walking, 5.6% and 9.6% for outdoor running, 9.7% and 13% for using an elliptical, 9.5% and 17.7% for biking, and was 26.9% for swimming.

**Conclusions:**

In a controlled setting, wearable activity trackers provide accurate recognition of the type of some common physical activities, especially outdoor walking and running and walking on a treadmill. The accuracy of measurement of activity duration varied considerably by activity type and tracker model and was poor for complex sets of activity, such as a run embedded within 2 walking segments.

## Introduction

### Background

Adequate physical activity participation is one of the most important behaviors people can adopt to maintain their health and well-being. Physical activity reduces the risk of several major chronic diseases [1] and early mortality [2], reduces health risks associated with overweight and obesity [3], and improves psychological outcomes, including mood and energy [4]. For adults, the 2018 American federal aerobic physical activity guidelines prescribe ≥150 min per week of moderate-intensity activity or ≥75 min of vigorous-intensity physical activity or an equivalent combination of the two [5]. Results from US national surveillance estimates show suboptimal rates of activity nationwide and highlight the importance of promoting physical activity participation to increase the overall health of the population [6].

Consumer-grade activity trackers are one tool that may help individuals increase and monitor their physical activity participation. These devices are available to consumers at a relatively low cost, with approximately 14 million Fitbits alone sold in 2018 [7] and 120 million devices projected to be sold by 2019 [8]. Such trackers have been shown to support increased physical activity participation in adults [9,10] and are suitable for incorporation into clinical research and health promotion interventions [9,11,12]. Naturally, the extent of the utility of these devices depends on the accuracy of their features. Peer-reviewed independent research is critical to understanding and validating these technologies, particularly as manufacturers typically neither provide comprehensive results of internal studies nor release their proprietary classification algorithms. Previous validation studies have reported high correlations between device step counts and the criterion [13,14] and a general underestimation of energy expenditure as compared with criterion measurements [15], and heart rate validation studies have shown that wearable devices are more accurate during rest than during moderate exercise [16-18].

With the evolution of physical activity algorithmic-based classification, manufacturers are developing beyond-the-manual logging of activities (eg, a user tagging an activity as *running* or *cycling*) toward use of pattern recognition algorithms to automatically detect certain activity types. Automatic activity recognition—called SmartTrack on Fitbit trackers [19] and Move IQ on Garmin trackers [20]—allows a wearable activity tracker to recognize and classify specific activity types, without input from the user. If accurate, this feature could enhance health-based research by lending insight into what types of activities participants are performing and what could aid researchers in tracking adherence to physical activity guidelines and fidelity to interventions.

### Objectives

Given the importance of understanding the accuracy of trackers’ new automatic activity detection capabilities, and the lack of current validation studies available on this feature, the objective of this study was to explore the accuracy of this feature on 4 commercially available wearable activity trackers in a controlled setting: the Fitbit Flex 2, the Fitbit Charge 2, the Fitbit Alta HR (Fitbit), and the Garmin Vívosmart HR (Garmin). Specifically, this study aimed to determine (1) the accuracy of the activity-type identification and (2) the accuracy of the measured duration of activity in a controlled setting.

## Methods

### Participants

This study protocol was approved by the University of Wisconsin-Madison Health Sciences Institutional Review Board. Written informed consent was obtained for all participants. Participants were adults aged from 18 to 50 years, who were able to perform moderate- and vigorous-intensity physical activity and were free from specific health limitations, including heart attack, angioplasty or heart surgery in the previous 3 months, active chest pain, shortness of breath, fainting, angina pectoris, current pregnancy, or any physical disability that would preclude use of a treadmill, an elliptical, or a bicycle. Participants were excluded from swimming if they reported an inability to swim front crawl (ie, freestyle) for 15 min. Participants were also excluded if their measured resting systolic blood pressure (via Riester ri-champion N automatic cuff, Riester) was > 180 mmHg or resting diastolic blood pressure was > 110 mmHg.

### Study Design

The study comprised 4 activity modules (Table 1) designed to reflect some of the most common types of moderate-to-vigorous intensity physical activity with the intention of evaluating 34 trials per module on the basis of previous validation studies with small sample sizes [15]. Power analyses were not conducted because of a lack of previously published (or *a priori)* identified effect sizes. Participants self-selected which module or modules they wished to complete. Participants could complete more than 1 module if desired, but each module was scheduled on a different day to avoid excessively long activity participation in a single day by each participant. Participation in multiple modules was not expected to influence results.

**Table 1:** Description and duration of activities in each of the experimental modules.

Module and activities		Activity duration
**Module A^a^**		
	Treadmill walk (2-4.5 mph)	15 min
	Treadmill run (>4.5 mph)	15 min
	Embedded run (walk, run, walk)^b^	25 min (5-min walk, 15-min run, 5-min walk)
**Module B^a^**		
	Outdoor walk	15 min
	Outdoor run	15 min
	Elliptical	15 min
**Module C**		
	Bike	15 min
**Module D**		
	Swim	15 min

^a^Activities in this module separated by 10 min of rest.

^b^These activities were completed at the same miles per hour (mph) designation as the previous activities. The purpose of the embedded run was to test the trackers’ ability to detect a run with walking before and after.

### Activity Modules

*Activity Module A* was completed on a treadmill (Trackmaster and Precor Inc) and comprised 15 min of walking (at speeds of 2 to 4.5 mph), 10 min of stationary rest, 15 min of running (>4.5 mph), 10 min of stationary rest, and a 15-min run embedded in 5-min segments of walking to simulate a warm up and cool down before and after a run (walk-run-walk). The purpose of the treadmill run embedded in walking bouts was to test the trackers’ ability to detect the running bout when preceded and followed by bouts of walking. This activity is referred to as the *embedded run*. *Activity Module B* comprised walking outdoors on a continuous path for 15 min, 10 min of stationary rest, running on a path for 15 min, 10 min of stationary rest, and using an elliptical trainer with moving arm handles (Precor Inc) for 15 min. Participants were instructed to use the moving arm handles on the elliptical machine for the activity’s duration. *Activity Module C* comprised 15 min of outdoor cycling on a continuous path (referred to as *bike* or *biking*). *Activity Module D* comprised 15 min of swimming in a 25-yard, 8-lane indoor pool. Participants began wearing devices following initialization in the laboratory; then they walked to the treadmill, pool, outdoor walking path, or other activity location (<5 min). Following each activity within the module, participants began the bout of sedentary rest. No sedentary rest was observed after the last activity in each module. Directly following each activity, participants reported a rating of perceived exertion (RPE) using the Borg RPE scale, which has high validity and reliability for reporting relative exercise intensity [21,22], to assess the intensity at which the trackers might successfully detect an activity and duration. Participants removed the trackers immediately after the last activity in each module.

### Devices

The 4 devices used in this study were the Fitbit Flex 2, the Fitbit Charge 2, the Fitbit Alta HR (Fitbit), and the Garmin Vívosmart HR (Garmin). These devices were chosen because at the time of the study, they were the only ones that offered the automatic activity detection feature. Participants wore all 4 devices concurrently, 2 on each wrist, and placement was randomized—although previous validation work [16] demonstrated that tracker accuracy does not differ by wrist location. For Module D, only the Fitbit Flex 2 was worn as the others do not detect swimming. Following informed consent, participants completed a brief health history form and a demographics questionnaire, and height and weight were measured once using a stadiometer (Health o meter, Welch Allyn). All trackers were initialized using their respective Fitbit or Garmin apps, using each participant’s actual height, weight, sex, and age. The same 4 devices were reinitialized and used for each participant in the study to minimize the potential risk of interdevice heterogeneity. The Fitbit devices were programmed to detect activities lasting longer than 10 min (the shortest amount of time required for activity-type recognition by the devices).

### Outcome Measures

Each activity performed was observed and recorded in conjunction with a timer on a laptop, which was used to record the duration of the activity; this was considered the criterion measure. The timer was stopped when the participant came to a complete stop at the end of the activity, and the duration was recorded. Upon completion of each module, the trackers were synced to their respective apps on a laptop, and the trackers’ designation of activity type and duration were recorded from the apps. A correct identification occurred when the tracker’s activity designation matched the actual activity performed. An erroneous identification occurred when the tracker’s activity designation did not match the actual activity performed, and a missed identification occurred when the tracker did not designate any activity when there was an activity performed.

### Statistical Analysis

All statistical analyses were completed using Statistical Analysis Software version 9.4 (SAS Institute Inc.) and Microsoft Excel version 1811 (Microsoft). Data from all users were inspected for errors before analysis. One participant’s elliptical trainer data were excluded because of failure to follow protocol (the participant did not use the moving arm handles). A total of 2 participants who were unable to complete the swimming activity were excluded from analyses. Descriptive statistics were used to characterize the participant sample and the mean Borg RPE of each activity. Frequency counts were calculated to analyze the number of correct identifications and the amount of missed and erroneous identifications. Summary statistics and box plots were produced to analyze the duration of activities that were correctly identified by the trackers. Mean absolute percent error (MAPE) (1/n*Ʃ| *true duration* – *device-based duration measure* |) was calculated for each activity and device to establish the differences between the criterion duration (duration measured with a laptop timer) and device measured duration.

## Results

Participants in this study (N=69) were 61% female, had a mean age of 26.4 (SD 8.7) years, and had an average body mass index of 23.9 (SD 4.1) kg/m^2^. Of the 69 participants, 36 (52%) participants chose to complete 1 module, 10 (15%) participants chose to complete 2 modules, 16 (23%) participants chose to complete 3 modules, and 7 (10%) participants chose to complete all 4 modules. Although the study was designed for 34 trials per module, the actual number of trials used in the analysis varied slightly because of infrequent but occasional inability to sync devices and retrieve data between study visits. The mean Borg RPE rating was 8.6 (SD 1.8) for the treadmill walk (corresponds to an *extremely light* to *very light* intensity); 13.6 (SD 1.9) for the treadmill run (*somewhat hard* to *hard* intensity); 14.2 (SD 1.6) for the embedded run (*somewhat hard* to *hard* intensity), 8.3 (SD 1.6) for the outdoor walk (*extremely light* to *very light* intensity), 13.7 (SD 1.4) for the outdoor run (*somewhat hard* to *hard* intensity), 13.0 (SD 1.7) for using an elliptical machine (*somewhat hard* intensity); 11.3 (SD 1.4) for outdoor biking (*light* to *somewhat hard* intensity); and 14.2 (SD 2.1) for swimming in a lap pool (*somewhat hard* to *hard* intensity).

### Activity Identification

**Figure 1:**
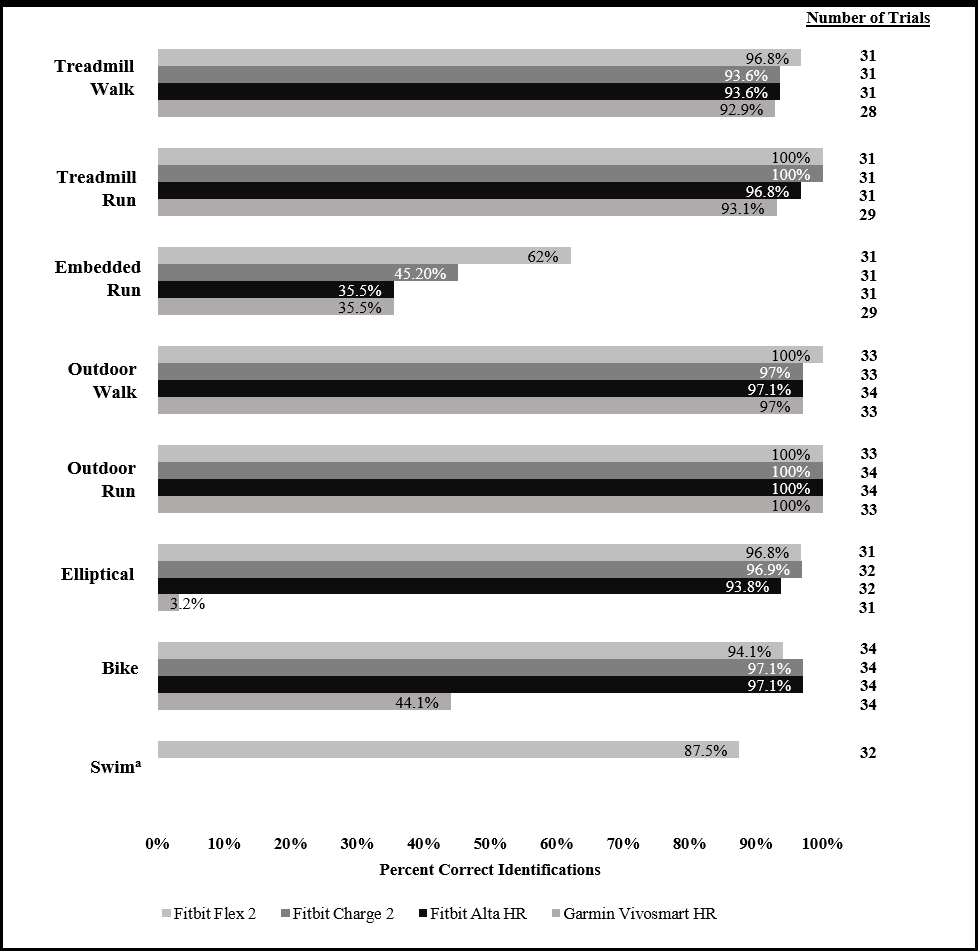
Percent correct identifications of eight activities by four wearable trackers.

Devices were highly successful in recognizing the simple bouts of walking and running on a treadmill or outdoors, struggled to recognize the embedded run, and had more variable success in recognizing the other activities (elliptical trainer, biking outdoors, and swimming; Figure 1). Overall, the activity with the highest proportion of correct identifications was outdoor running, which was detected for 100% of trials on all devices. Outdoor walking had a rate of correct identifications between 97% and 100%, dependent on tracker brand and model. Although slightly lower, both treadmill walking and running had rates of identification above 92%. Treadmill walking was correctly detected between 93% and 97% of trials dependent on tracker brand and model, and treadmill running was correctly detected between 93% and 100% of trials. The embedded run was detected less often between 36% and 62% of trials. As we were interested in the trackers’ ability to detect a run when it was preceded or followed by a short bout of walking, the correct designation for this activity was *run*.

Elliptical use was correctly detected in 91% to 93% of trials for Fitbit devices, but only 3% of trials by the Garmin device. Similarly, biking had a high rate of correct identifications by the Fitbit devices, between 94% and 97% of trials, and a lower recognition rate of 44% of trials by the Garmin device. Swimming was correctly detected for 88% of trials on the Fitbit Flex 2 device. Although we requested the use of front crawl stroke for the duration of the swim module, 5 participants switched to other strokes during the swim. When these participants were excluded from the analysis, the Fitbit Flex 2 had a correct recognition rate of 85% for 27 trials.

The types of observed misclassifications are shown in a confusion matrix (Table 2). A total of 9 misclassifications occurred for the Garmin device during the elliptical activity, the most misclassifications of any activity. A smaller number of misclassifications were observed for treadmill walking and running, outdoor walking, and swimming. The 2 misclassifications that occurred for the outdoor walking activity were misclassified for the same participant.

**Table 2:** Confusion matrix of activity identifications by actual activity type and device.

Activity and device		Identifications		Misclassified as a
		Correct	Erroneous	
**Treadmill walk**				
	Fitbit Flex 2	30	1	Run (1)
	Fitbit Charge 2	31	0	—^a^
	Fitbit Alta HR	31	0	—
	Garmin Vívosmart HR	28	0	—
**Treadmill run**				
	Fitbit Flex 2	31	0	—
	Fitbit Charge 2	31	0	—
	Fitbit Alta HR	30	1	Aerobic workout (1)
	Garmin Vívosmart HR	29	0	—
**Embedded run**				
	Fitbit Flex 2	31	0	—
	Fitbit Charge 2	31	0	—
	Fitbit Alta HR	31	0	—
	Garmin Vívosmart HR	29	0	—
**Outdoor walk**				
	Fitbit Flex 2	33	0	—
	Fitbit Charge 2	32	1	Elliptical (1)
	Fitbit Alta HR	33	1	Elliptical (1)
	Garmin Vívosmart HR	33	0	—
**Outdoor run**				
	Fitbit Flex 2	33	0	—
	Fitbit Charge 2	34	0	—
	Fitbit Alta HR	34	0	—
	Garmin Vívosmart HR	33	0	—
**Elliptical**				
	Fitbit Flex 2	31	0	—
	Fitbit Charge 2	32	0	—
	Fitbit Alta HR	32	0	—
	Garmin Vívosmart HR	22	9	Fitness (9); Other (6); Run (2)
**Swim**	
	Fitbit Flex 2	28	4	Aerobic workout (2); Walk (2)

^a^No activity misclassifications for this device/activity combination.

### Duration Accuracy

**Figure 2:**
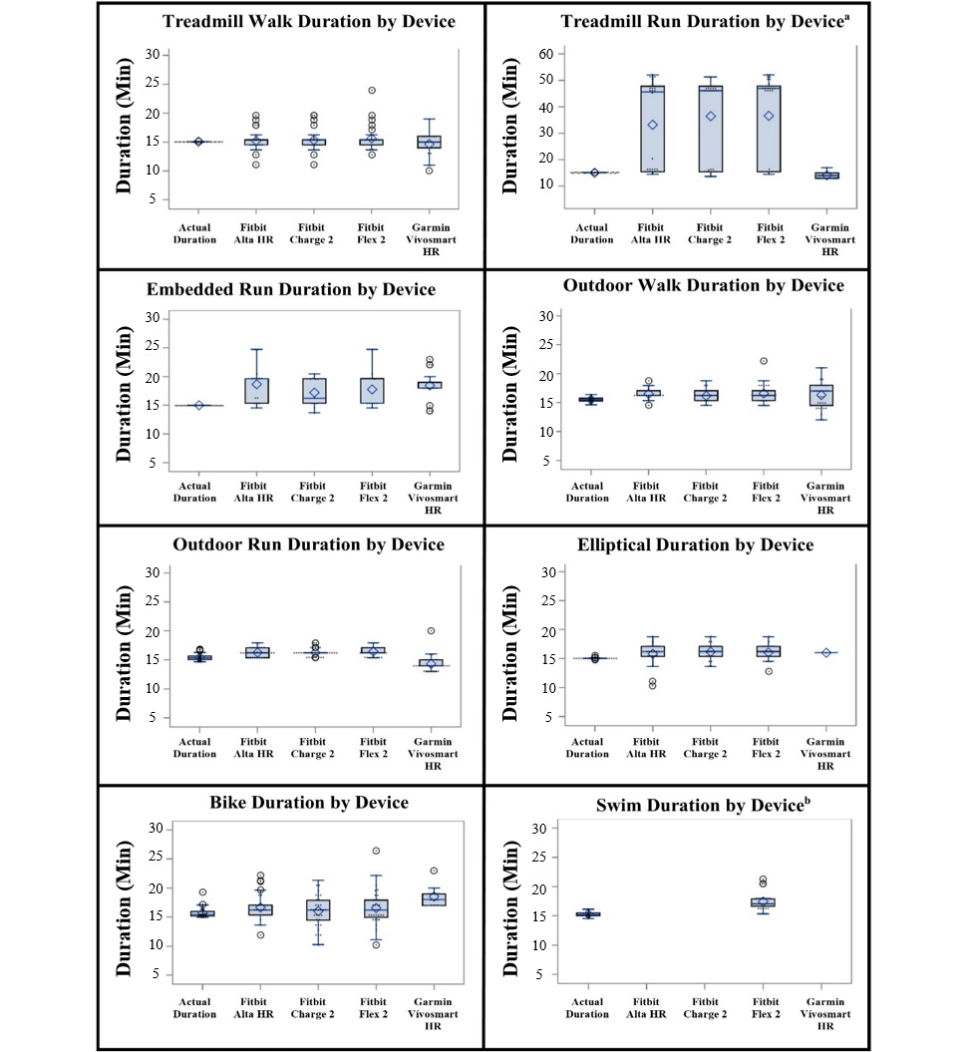
Box plots of the actual duration of each activity, compared to the duration estimated by the automatic activity recognition feature on each of the four trackers.

Results of the duration accuracy analysis show that detected mean duration was less than 4 min over or under the actual activity duration, except from the treadmill run for all Fitbit trackers, which was overestimated by a mean of 20 min (Figure 2). As the activity bouts were relatively short, even a few minutes of deviation in measurement of activity duration resulted in a substantial MAPE (Table 3). MAPEs were lower for all devices for treadmill walking (7% to 7.9%), outdoor walking (4.9% to 11.8%), and outdoor running (5.6% to 9.6%) than other activities, including treadmill running (8.7% to 144.8%), the embedded treadmill run preceded and followed by a walk (23.6% to 28.9%), an elliptical trainer (9.7% to 13%), biking (9.5% to 17.7%), or swimming (26.9%). Box plots for recorded duration show a spread in time for activities that were completed outdoors or in the pool, as participants needed to return to the activity start site (outdoors) or to the pool edge at the end of the activity. Thus, actual time fluctuated slightly around 15 min. All devices estimated treadmill walking within 1 min of the actual duration.

The Garmin device underestimated treadmill running duration by approximately 1 min. The embedded run duration was overestimated by a mean of 3 min by all devices. Outdoor walking was overestimated by a mean of 1 min by all devices. Outdoor running was overestimated by a mean of 1 min by Fitbit devices and was underestimated by about 1 min by the Garmin tracker. Using an elliptical and biking were overestimated by a mean of 1 min by all devices. Swimming was overestimated by approximately 2 min by the Fitbit Flex 2. The Garmin device was the only wearable tracker to underestimate activity duration, and when activity was overestimated by other devices, it was overestimated by less than 4 min on average by all devices, except for the treadmill run, which was overestimated by a mean of 20 min.

**Table 3:** Mean absolute percent error of duration measures by device and activity.

Activity type	Device			
	Fitbit			Garmin Vívosmart HR
	Flex 2	Charge 2	Alta HR	
Treadmill walk	7.9	7	7.6	7.7
Treadmill run	139.4	115.4	144.8	8.7
Embedded run	23.6	24.8	28.9	23.9
Outdoor walk	8.1	6.9	4.9	11.8
Outdoor run	6.6	5.6	5.6	9.6
Elliptical	9.7	10.2	10.2	13
Bike	13.3	9.5	12.9	17.7
Swim	26.9	—^a^	—	—

^a^No data acquired as Only the Fitbit Flex 2 is waterproof and can measure swimming.

## Discussion

### Principal Findings

The most important findings of this study are that the trackers correctly identified most of the common activity types tested in a controlled setting, especially walking and running, and that the trackers had variable success in identifying the duration of the performed activities. This study measured the accuracy of the automatic activity-type recognition and duration feature on 4 wearable activity trackers during walking, running, using an elliptical trainer, biking, and swimming. Although there are countless permutations of possible physical activity behavior (eg, combinations of intensity, duration, setting, and stops and starts), the goal of this study was to conduct a preliminary laboratory-based test of multiple common activity types. All tested trackers had better recognition of ambulatory-based activities such as walking or running as opposed to biking, using an elliptical, or swimming. This finding is consistent with previous validation studies that show wearable activity trackers generally have high correlations of step counts when compared with the criterion during ambulatory activity [23]. Although this study did not aim to validate step counts (which have already been well studied) [23], walking and running were the only activities tested that involved stepping, which may bolster the trackers’ detection capability.

Outdoor walking was misclassified as using an elliptical once by both the Fitbit Charge 2 and Fitbit Alta HR. These misclassifications occurred for the same participant and may be because of the participant having an arm movement pattern that mimics an elliptical arm handle pattern during walking. The high misclassification rate of using an elliptical by the Garmin device is concerning and indicates that it is likely unsuitable for apps where participants or patients may engage in use of elliptical machines as a regular part of their exercise regimen. With regard to swimming, although the Fitbit brand recommends swimming freestyle consistently for best detection results, it also claims the Fitbit Flex 2 can measure the backstroke, breaststroke, and butterfly [24]. Although we did not aim to measure strokes other than freestyle, it should be noted that the device did correctly identify all 5 of the swim trials that experienced a switch in stroke. This is important as stroke switching represents a more natural pattern than swimming a single stroke for an entire bout of activity, suggesting the tracker may be robust for capturing mixed-stroke swimming. However, because of the very small number of trials in which multiple strokes are used, additional data are needed to confirm whether this is true.

The detected mean duration was less than 4 min over or under the actual duration of the activity for outdoor walking and running, the embedded run, using an elliptical, outdoor biking, and swimming. Of these activities, MAPEs were the lowest for outdoor walking and running and treadmill walking. The treadmill run duration for all Fitbit trackers was considerably overestimated by an average of 15 min. The MAPE for treadmill running was also noticeably larger than other activities falling between 115.4% and 144.8%. The considerable overestimation was likely because of the trackers failing to recognize the 10 min of stationary rest after the treadmill run and including some duration of the embedded run (walk, run, walk). The addition of time occurred during 16 trials detected by the Fitbit Alta HR, 20 trials detected by Fitbit Charge 2, and 20 trials detected by the Fitbit Flex 2. If confirmed by future studies, this may indicate that trackers need improvement before they are suitable for satisfactorily identifying and measuring exercise sessions comprising intermittent or combined activities. This is a particular concern as many free-living activities are performed in short bouts and adjacent to other movements; thus, our findings suggest that at least for short bouts of activity (approximately 15 min), current trackers are prone to substantial error in duration estimates.

Similar results were observed for most activities by the Fitbit devices, regarding both activity detection and duration. The 3 Fitbit devices yielded activity detection results that were at most 3.2% different from one another for any ambulatory activity, excluding the embedded run, and had identical results for the outdoor run. This similarity is also observed for the nonambulatory activities. The Fitbit devices yielded results that were, at most, 3.1% different from one another (at least 0.1%) for using an elliptical and, at most, 3% different or identical for outdoor biking. When comparing the Fitbit brand MAPEs, the treadmill run activity shows the most variability in results with the Fitbit Alta HR and Fitbit Flex 2 differing by 29.4% and with all other activities having identical results or differing by, at most, 5.3%. It is likely that these devices use similar, if not identical, classification algorithms, as they are developed and manufactured by the same company. It is important to consider that future Fitbit devices may continue to yield similar results across devices. If the use of identical technology across devices is confirmed by the company, this may eliminate the need for testing multiple devices in a single study.

This paper provides, to our knowledge, the first data on the automatic activity-type recognition feature in wearable trackers. A major strength is the inclusion of multiple common activities in different contexts (eg, walking outdoors and on a treadmill). The use of a standardized protocol is both a strength and a limitation. As no previous data on this feature are available, we chose a standardized protocol because it provides the ability to directly observe the type and duration of activity participation. However, it is unknown how well the results of this structured experimental study would generalize to free-living settings. Individuals who use wearable activity trackers tend to wear them all day, during which a wide variety of movements and activities are performed. By contrast, in a laboratory-based protocol, the devices are worn only during the study visit and are removed after completion of the final activity in each module. This removal may aid the tracker in determining the stop time of those activities. A second limitation is that the we did not attempt to determine how the devices’ accuracy might be affected by the duration, speed, or intensity of an activity. Our goal was to test multiple activity types, both indoor and outdoor, to provide a preliminary test of the activity recognition function. Although it is never possible to test all possible combinations of activity type, duration, setting, speed, and intensity, additional research will provide substantial insight on how these factors generally affect accuracy of the automatic activity recognition feature.

Our results add to the evidence base of previous validation studies that have examined the accuracy of other wearable tracker features, such as steps, which show high correlation between criterion and device [13,14], energy expenditure, which is generally underestimated when compared with the criterion [15], and heart rate, which is more accurate at rest than while exercising [16-18]. Although wearable trackers do not correctly measure all activity metrics perfectly for every trial, their ability to provide objective measures of some aspects of activity is a great advancement from even 10 years ago, with technology likely to improve rapidly with time.

### Conclusions

Wearable activity trackers correctly identified the type of isolated bouts of some common physical activities, although accuracy decreased substantially for certain activities that were adjacent (or nearly so) with one another, and 1 device had very poor detection on the elliptical trainer. Future directions for research on wearable trackers include testing the recognition capabilities of more and complex permutations of activity participation, including aerobic workouts, such as kickboxing or high intensity interval training, sports such as basketball and soccer, slower walking or light-intensity activities (of particular interest for chronic disease populations and older adults), intermittent activities such as jogging interrupted by stoplights, and other free-living activity patterns at a variety of exercise intensities. Owing to the nature of the rapidly evolving wearable technology market, future directions also include testing the automatic activity detection capability in new devices as they are released to the public.

## Abbreviations

MAPE: mean absolute percent error
RPE: rating of perceived exertion
